# Fusion beat in patients with heart failure treated with left ventricular pacing: may ECG morphology relate to mechanical synchrony? A pilot study

**DOI:** 10.1186/1476-7120-6-1

**Published:** 2008-01-01

**Authors:** Lorella Gianfranchi, Katia Bettiol, Biagio Sassone, Roberto Verlato, Giorgio Corbucci, Paolo Alboni

**Affiliations:** 1 Division of Cardiology, Ospedale di Cento (Fe), via Vicini 2, Cento, Italy; 2 Ospedale Bentivoglio, Via G. Marconi 35, 40010 Bentivoglio(Bo), Italy; 3 Ospedale Camposampiero, Via P. Cosma 1, 35012 Camposampiero (Pd), Italy; 4 Vitatron Medical Italia, Milano, Italy; 5 Responsible of EP laboratory, Division of Cardiology, Ospedale di Cento (Fe), via Vicini 2, 44042, Cento, Italy

## Abstract

**Background:**

Electrical fusion between left ventricular pacing and spontaneous right ventricular activation is considered the key to resynchronisation in sinus rhythm patients treated with single-site left ventricular pacing.

**Aim:**

Use of QRS morphology to optimize device programming in patients with heart failure (HF), sinus rhythm (SR), left bundle branch block (LBBB), treated with single-site left ventricular pacing.

**Methods and Results:**

We defined the "fusion band" (FB) as the range of AV intervals within which surface ECG showed an intermediate morphology between the native LBBB and the fully paced right bundle branch block patterns.

Twenty-four patients were enrolled. Echo-derived parameters were collected in the FB and compared with the basal LBBB condition. Velocity time integral and ejection time did not improve significantly. Diastolic filling time, ejection fraction and myocardial performance index showed a statistically significant improvement in the FB. Interventricular delay and mitral regurgitation progressively and significantly decreased as AV delay shortened in the FB. The tissue Doppler asynchrony index (Ts-SD-12-ejection) showed a non significant decreasing trend in the FB. The indications provided by the tested parameters were mostly concordant in that part of the FB corresponding to the shortest AV intervals.

**Conclusion:**

Using ECG criteria based on the FB may constitute an attractive option for a safe, simple and rapid optimization of resynchronization therapy in patients with HF, SR and LBBB.

## Introduction

Patients with heart failure (HF), low ejection fraction (EF) and left bundle branch block (LBBB) can be treated with cardiac resynchronization therapy (CRT). CRT is an adjunctive treatment currently indicated for patients remaining symptomatic in NYHA class III or IV despite optimal medical therapy [1-3].

Many observational studies, as well as a series of randomized, controlled trials have demonstrated the safety, efficacy and long-term beneficial effects of CRT. These studies have shown statistically significant improvements in quality of life, NYHA functional class ranking, exercise tolerance [4-8] and left ventricular reverse remodelling [8-10]. In addition, reductions in mortality [8] and hospitalization have recently been demonstrated [4,5,7,8].

Most of the available clinical data have been derived from studies involving permanent biventricular pacing (BIVP). Single-site left ventricular pacing (LVP) confers similar improvements in clinical parameters to BIVP [11-13], even in the long term [14-16]. In comparison with BIVP, single-site LVP is associated with equivalent or better haemodynamic improvement, even during physical exercise [17].

In patients with left ventricular conduction delay and sinus rhythm, LVP alone significantly increases LV systolic function. Although indications for LVP must still be clear defined, there is growing evidence suggesting that applying LVP is comparable with the BIVP mode in selected HF patients presenting LBBB. Two multi-centre randomized trials [18,19] have confirmed that there are no substantial differences in response between the two pacing modes. The present availability of devices with separate channels allows the application of LVP while ensuring pacemaker or ICD back-up on the right ventricular lead. The recent European guidelines on cardiac pacing and CRT [20] state that in selected patients with LBBB and conventional indication to CRT, is reasonable to consider LVP alone.

Electrical fusion between LVP and spontaneous right ventricular activation is considered the key to resynchronisation in sinus rhythm patients treated with single-site LVP [21-26]. Depending on the pacing site and the atrioventricular (AV) interval programmed, atrial synchronous LVP leads to electrical fusion between the intrinsic excitation of the septum and right ventricle, and the stimulated premature excitation of the left ventricular free wall. The degree of electrical coordination depends on the conduction velocity of the intrinsic AV conduction, the prematurity of LVP, LV transmural activation time, the time needed for the paced stimulus to break through into the endocardium, and on the propagation velocity within the myocardial and subendocardial layers.

Electrical fusion can easily be detected through surface ECG: a simple morphological analysis may advise how to programme the device. The aim of this pilot study was to evaluate the use of the QRS morphology of fusion beats to optimise programming in CRT patients with sinus rhythm, LBBB and single-site LVP.

## Methods

Consecutive patients underwent CRT device implantation in accordance with current guidelines. During post-implantation (>1 month) echocardiographic evaluation, the device was tracked by spontaneous atrial activity thanks to a preserved sinus node function. Ventricular pacing was enabled only in the left ventricle.

On shortening the programmable AV interval by 10 ms steps, we obtained a series of 12-lead ECG showing progressive transition in morphology from LBBB to a completely left preexcited right bundle branch block (RBBB), passing through intermediate QRS of "fusion". The transition was most easily detected in lead V1.

We defined the "fusion band" as the range of AV intervals at which surface ECG showed an intermediate morphology between the native LBBB pattern and the fully paced RBBB pattern.

**Figure 1:**
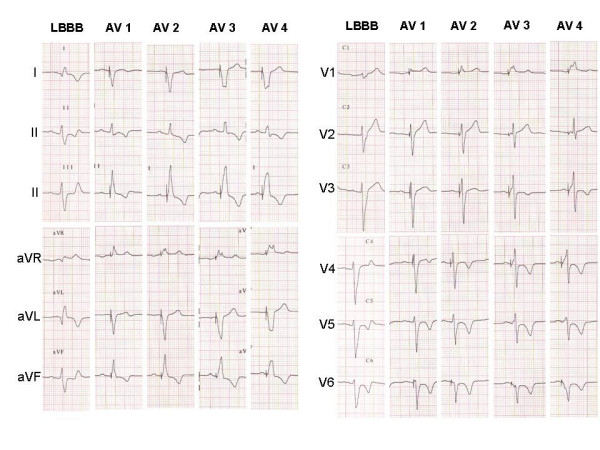
The upper limit of the band was set at an AV interval (AV1) 40 ms shorter than the intrinsic conduction time (time between atrial and right ventricular sensing), a choice that gives LBBB morphology but with a slight variation from that of the native QRS. The lower limit was set at the AV interval (AV4) that produced an RBBB-like morphology, but not complete RBBB. The time between the limits was divided into three intervals, thus identifying two intermediate AV intervals: AV2 and AV3.

We chose to set the upper limit of the band at an AV interval 40 ms shorter than the intrinsic conduction time (time between atrial and right ventricular sensing), a choice that gives LBBB morphology but with a slight variation from that of the native QRS; the lower limit was set at the AV interval that produced an RBBB-like morphology, but not complete RBBB. The time between the limits was divided into three intervals, thus identifying two intermediate AV intervals (fig 1).

Patients underwent a conventional echocardiographic examination including M-mode, two-dimensional and Doppler analysis to determine LVEF by means of the biplane Simpson's equation (Vivid 7, GE Vingmed Ultrasound AS, Horten, Norway). Images were obtained during stable haemodynamic conditions by using a 3.5-MHz transducer in standard left parasternal and apical views. The following variables were calculated at each of the patient-specific predefined AV intervals, as mentioned above: LV diastolic filling time (DFT) was measured on the pulsed-wave Doppler of mitral inflow as the time interval from the onset of the E wave to the end of the A wave; mitral regurgitation (MR) was visualized by colour Doppler flow imaging in the 4-chamber apical view and its degree expressed as the percentage of the regurgitant jet area relative to the left atrial size [27]; acute changes in LV systolic performance were indirectly estimated as either the velocity-time integral (VTI) or the ejection time (ET) calculated on the aortic continuous-wave Doppler spectrogram; to investigate the global LV function, the myocardial performance index (MPI) was also measured [28,29]. The interventricular mechanical delay was measured on the pulsed-wave Doppler of aortic and pulmonary outflow as the time interval from the onset of the QRS to the beginning of the aortic and pulmonary flow velocity, respectively. Tissue Doppler imaging (TDI) was performed to assess LV dyssynchrony during an off-line analysis of stored images obtained by means of apical 4-chamber, 2-chamber and long-axis views (EchoPac 6.1, GE Vingmed Ultrasound AS). An LV 12-segment model (6 basal and 6 mid segments) was used and LV synchronicity was derived from the standard deviation (SD) of the averaged T_s _of all 12 LV segments [30]. All echocardiographic measurements were performed by the same operator and averaged over 3 consecutive cardiac cycles during stable sinus rhythm.

The study had the approval of the Ethics Committee of the Azienda Unità Sanitaria Locale di Ferrara, Via Cassoli 30, 44100 Ferrara (Italy), and informed consent to participate in the investigation was obtained from each patient before enrolment.

### Statistical analysis

All data are expressed as mean ± SD. An intra-patient analysis was performed and the data were compared by means of paired, 2-tailed Student's t-Test. A P value <0.05 was regarded as significant.

## Results

We enrolled 24 consecutive patients with HF, sinus rhythm and LBBB, in whom a CRT device had been implanted. Table 1 shows the baseline characteristics of the patient population.

**Table 1:** Patient Population (24 Pts)

Age, yrs	66 ± 10
Males, N (%)	15 (63%)
Females, N (%)	9 (37%)
QRS width, ms	166 ± 17
NYHA class, N	2.73 ± 0.46
EF, %	28 ± 5
Post_MI Cardiopathy, N (%)	16 (67%)
Valvular Cardiopathy, N (%)	1 (4%)
Idiopathic Dilated Cardiomyopathy, N (%)	7 (29%)

NYHA = New York Heart Association; EF = ejection fraction; MI = myocardial infarction.

The fusion band, as assessed through surface ECG analysis, ranged from 95 ± 31 to 189 ± 45 ms. Mean basal QRS width of the patients was 166 ± 17 ms.

**Figure 2:**
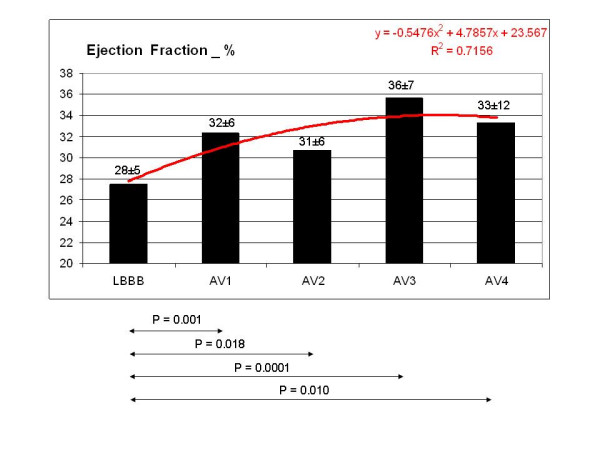
EF in the five tested configuration: EF shows a slight but statistically significant increase in the fusion band compared to the basal LBBB pattern. The increment in the fusion band ranges from 11 to 29%.

LVEF had a slight but significant increase in the fusion band compared to LBBB (fig 2).

**Figure 3:**
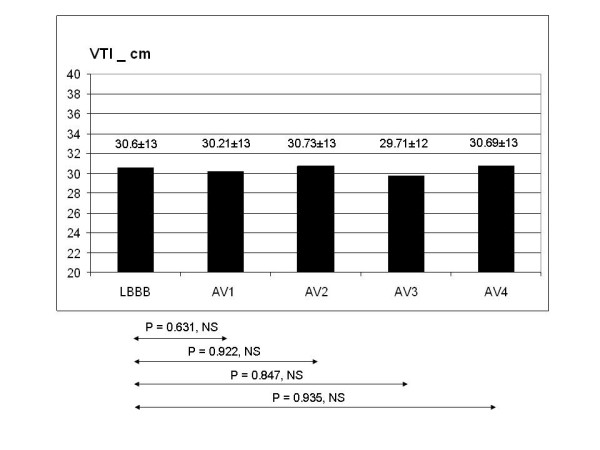
VTI neither changed significantly nor showed a trend in the tested configurations. The maximum value corresponds to AV2 but it is not clinically significant compared to the LBBB condition.

**Figure 4:**
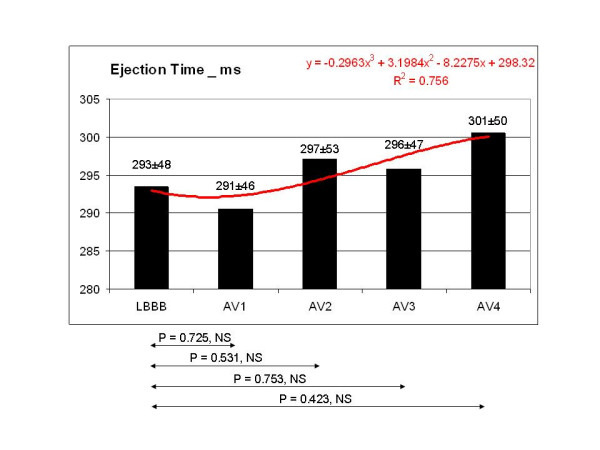
Compared to the LBBB pattern, ET showed a tendency to increase at the shortest AV delays in the fusion band, but without reaching statistically significant values.

**Figure 5:**
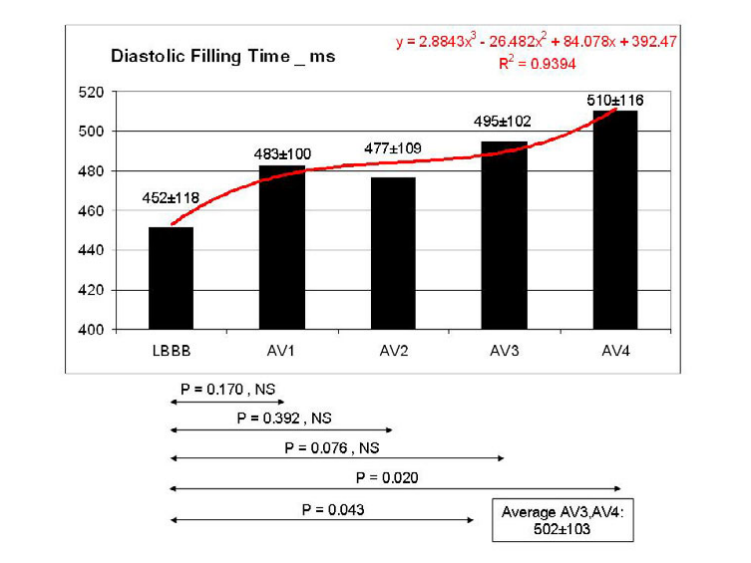
DFT showed a statistically significant increase corresponding to AV 4, the shortest AV interval. The mean value of DFT corresponding to AV3 and AV4 was also statistically significant compared to the LBBB pattern, showing an increase >10%.

**Figure 6:**
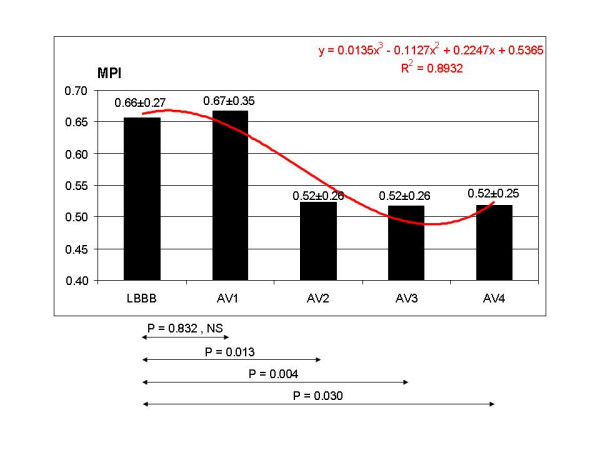
MPI showed a statistically significant decrease at AV2, AV3, AV4, but not in AV1. The decrease in AV2, AV3 and AV4 was >20% compared to the LBBB pattern.

**Figure 7:**
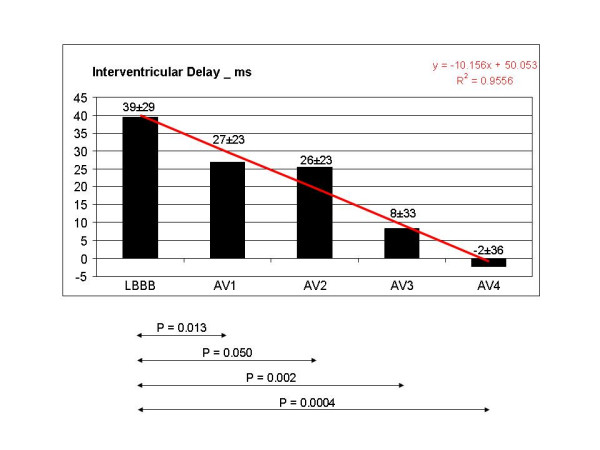
Interventricular delay progressively and significantly decreased as AV delay shortened: the best clinical values corresponded to the shortest AV delays AV3 and AV4, showing a reduction of 79% and 105% respectively.

**Figure 8:**
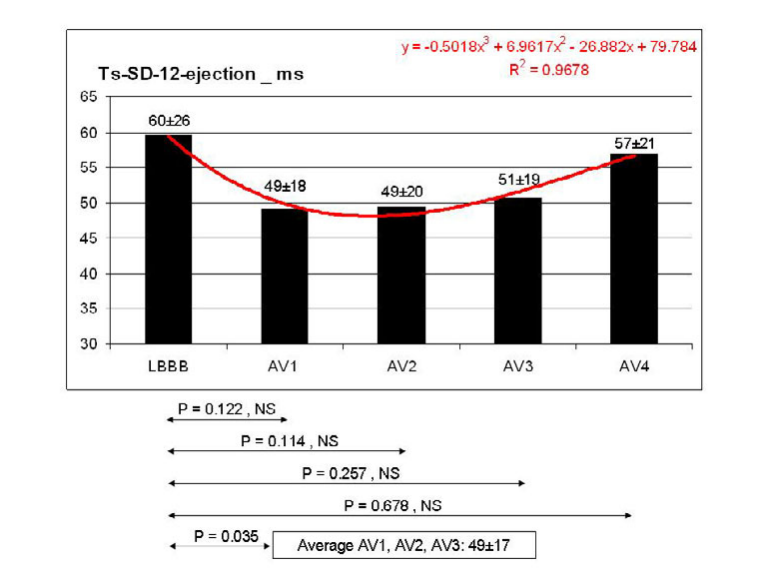
The TDI asynchrony index (Ts-SD-12-ejection) did not vary significantly between each of the different steps and the value recorded during LBBB, but it showed a decreasing trend at AV1, AV2 and AV3. The mean value of Ts-SD-12-ejection corresponding to AV1, AV2 and AV3 reached statistical significance compared to the LBBB pattern with >18% improvement.

**Figure 9:**
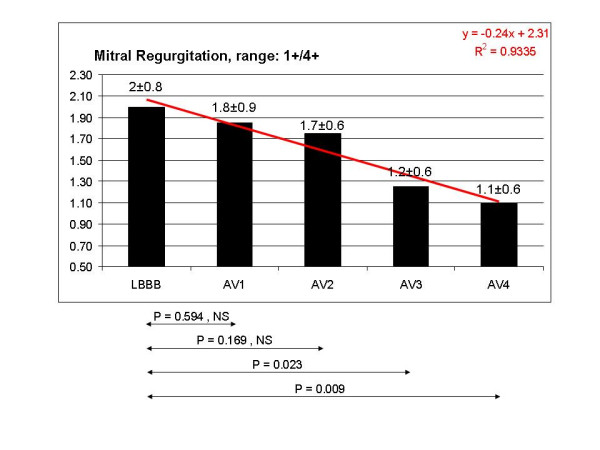
Mitral regurgitation showed a tendency to reduce in the initial part of the fusion band, and was significantly lower at the two shortest AV intervals AV3 and AV4, with a reduction of 40% and 45% respectively compared to the LBBB pattern.

In comparison with the pattern observed during LBBB, VTI (fig 3) and ET (fig 4) did not show any statistically significant variation in the 4 configurations tested, even though ET showed a tendency to increase at the shortest AV delays in the fusion band. By contrast, DFT showed a statistically significant improvement, corresponding to AV4. The mean value of DFT corresponding to AV3 and AV4 was also statistically significant compared to LBBB (fig 5). MPI showed a statistically significant decrease at AV2, AV3, AV4 (fig 6). Interventricular delay progressively and significantly decreased as AV delay shortened: the best clinical values corresponded to the shortest AV delays (fig 7). The TDI asynchrony index (Ts-SD-12-ejection) did not vary significantly between each of the different steps and the value recorded during LBBB, though it did show a decreasing trend at AV1, AV2 and AV3. The mean value of Ts-SD-12-ejection corresponding to AV1, AV2 and AV3 reached statistical significance in comparison with the basal LBBB (fig 8). Mitral regurgitation showed a tendency to reduce in the initial part of the fusion band, and was significantly lower at the two shortest AV intervals (fig 9).

## Discussion

To the best of our knowledge this pilot study is the first study to utilize the well-known electrical concept of fusion to guide the optimization of CRT in patients treated with single-site LVP. On the surface electrocardiogram, electrical fusion generates variation in the QRS complex morphology. In our study, we programmed AV intervals in such a way as to modulate the degree of fusion between spontaneous septum/right-ventricular activation and artificial left ventricular stimulation, while keeping the patient's spontaneous atrioventricular conduction unaltered. Optimization of the AV interval, as intended in patients with complete AV block, was not the aim of our study.

CRT is an electrical approach that uses pacing and electrophysiologic principles to improve mechanical performance. When only the LV is stimulated, the interventricular conduction time from left to right allows normal conduction to occur over the right bundle at the longest AV intervals. This results in the fusion of LV pacing and intrinsic conduction, thereby producing biventricular activation by means of single-site pacing.

Initially, the role of intrinsic conduction through the right bundle branch on the haemodynamic effect of left ventricular stimulation was studied in an animal model [23]. The findings of that study indicated that, in canine hearts with experimental LBBB, LV-based pacing can restore LV function in terms of LV dP/dtmax and Systolic Work. Maximum improvement of LV function is consistently obtained on intra-ventricular resynchronization of activation. This requires pacing with AV delays equal to the baseline PQ time. Another experimental study [25] showed that, at intermediate AV delays, the electrical activation pattern led to a significant reduction in both interventricular and left intraventricular electrical asynchrony.

Subsequently, the relationship between fusion and mechanical efficiency was acutely evaluated in CHF patients by measuring dP/dt [24]. In that study, LV pacing proved superior to BIV pacing, provided that it was associated to ventricular fusion.

While QRS duration is used as a criterion for patient selection, QRS narrowing in response to CRT has proved to be a disappointing index of haemodynamic improvement.

As fusion induces mechanical resynchronization, QRS morphology, rather than duration, may be utilized to identify the desired re-homogenisation.

Recently van Gelder [31] proposed a method to programme the optimal atrial sensed AV interval as determined from the QRS morphology obtained at the optimal atrial paced AV interval in patients with normal AV conduction and BIVP. This study represents a further confirmation that the morphology of the QRS complex is determined by the activation sequence of the ventricles and identical morphologies represent identical activation sequences. In patients with BIVP, QRS morphology depends on fusion between three propagation waves due to right ventricular pacing, LVP, and intrinsic right bundle conduction [31].

In patients with single-site LVP, ECG morphology depends on fusion of only two propagation waves due to LVP and intrinsic right bundle conduction. In this setting QRS morphology may provide a simple guide to the proper programming of the device, as progression in the degrees of fusion appears on the surface ECG as variation in the QRS complex [32].

As it is still unclear which is the most reliable and reproducible echo parameter for evaluating CRT patients, we chose a set of parameters that quantify different aspects of cardiac function in order to explore the different levels of dyssynchrony that may be present in failing hearts. Similarly, it is still unclear whether the optimal haemodynamic effect of CRT coincides with maximal reduction in interventricular or intraventricular asynchrony [9,23,26,33,34]. Many of the parameters we tested showed a statistically significant improvement, at least in a part of the fusion band, compared with native LBBB: MPI, DFT, interventricular delay, mitral regurgitation and TDI index. ET did not reach significant variations but showed a favourable trend along the fusion band, again in comparison with the basal LBBB. VTI was roughly constant. While all the aspects of cardiac function examined were better in the fusion band than during native LBBB, it was not possible to identify an AV delay at which there was full concordance among the various parameters.

**Figure 10:**
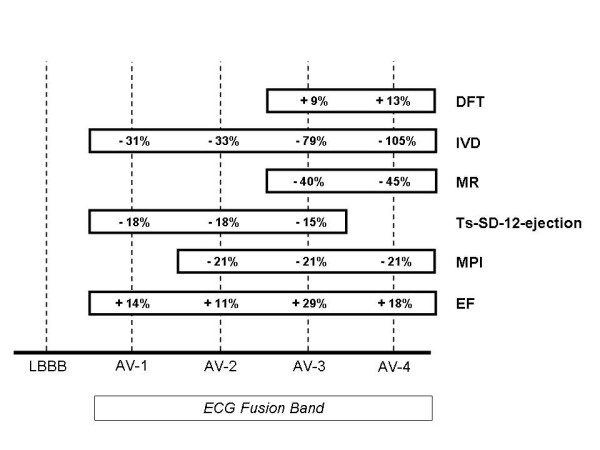
This figure summarizes the indications provided by parameters that showed a statistically significant improvement in the corresponding tested configurations. The variations (%) are shown for each parameter with reference to the basal condition of LBBB. VTI and ET were excluded because they did not show any statistically significant improvement in any of the tested AV intervals. DFT significantly increased at AV3 and AV4. Interventricular delay (IVD) statistically decreased at every tested configuration in the fusion band, even if the optimal clinical values corresponded to AV3 and AV4. MR showed a significant and clinically important reduction at AV3 and AV4. Ts-SD-12-ejection improved at AV1, AV2 and AV3 and the corresponding mean value was statistically significant compared to LBBB. MPI significantly decreased at AV2, AV3 and AV4. EF significantly increased in the fusion band with the best values corresponding to AV3 and AV4. In accordance with these indications it seems reasonable to programme single-site LV pacing at the AV intervals within the fusion band, mainly within the shorter half of it. Indeed, setting a value of AV interval inside the fusion band improves cardiac function in comparison with LBBB. At the shortest AV intervals of the band there is the largest overlapping among the indications provided by the measured parameters.

In the real world, programming devices usually means choosing the "best" value of one or more parameters, which are analyzed acutely, basically at rest, as we did in this pilot study. On the basis of our observations, it seems reasonable to programme single-site LV pacing at the AV intervals within the predefined band, mainly within the shorter half of the band, as is clearly shown in fig 10; indeed, setting a value of AV interval inside the fusion band improves cardiac function in comparison with LBBB. It's even clear that a setting that optimizes one parameter is not ideal for another one: probably electrical therapy cannot optimize all mechanical parameters in a homogeneous fashion and this should be taken into account when managing patients treated with CRT.

In the shortest part of the band, we observed the largest overlapping among the parameters. Moreover, setting an AV value inside the fusion band may guarantee the persistence of fusion, and thus of the haemodynamic benefits, even during the continuous variation of spontaneous atrioventricular conduction, and even during physical exercise.

Naturally, the approach based on the ECG detection of the fusion band can be adopted only in sinus rhythm patients, as they provide a reliable trigger, in terms of atrial activity, for LV pacing. When possible, the use of single-site LV pacing enables the life of the implanted device to be significantly prolonged.

This approach does not obviate the need for the RV lead, as only patients with LBBB and stable sinus rhythm can benefit from fusion with spontaneous RV activation, and therefore do without RV pacing. Data from the literature show that during atrial fibrillation (AF), provided that LV pacing is not inhibited by high rates, BIV pacing seems superior to single-site LV pacing [35,36]. As previously documented or new-onset AF is frequent in HF patients, the presence of the RV lead could allow BIV pacing during AF episodes. In this respect, technology could provide us with an algorithm to switch from LV pacing to BIV during AF.

Moreover, patients in whom an implantable defibrillator is indicated need an RV lead for appropriate sensing and defibrillation of the arrhythmia in any case.

Finally, we did not compare LVP with BIVP because this was not the aim of this pilot study. Our objective was to find out an ECG based method to optimize LVP. It seems reasonable that a similar method may be applied even to optimize BIVP, as fusion band may be identified also with this pacing mode.

## Limitations

As patients were evaluated at rest, the study yielded no data regarding the dynamic behaviour of the fusion band during exercise stress testing.

We compared the parameters recorded in the fusion band with native LBBB, but we did not evaluate the extreme condition of complete RBBB corresponding to very early depolarization of the left ventricle. In practice, this evaluation is feasible only in patients with a relatively long PR interval, in whom the programmable AV intervals could be short enough to obtain full capture of the entire myocardium through left pacing, thereby anticipating the spontaneous right bundle branch conduction.

Finally, we did not compare subgroups of patients with ischemic and non-ischemic cardiopathy, owing to the relatively low number of patients in these subgroups.

## Conclusion

Using ECG criteria based on the "fusion band" method to optimise single-site LV stimulation seems feasible and reliable. It may constitute an attractive option for the safe, simple and rapid management of devices implanted in patients with heart failure, sinus rhythm and left bundle branch block. Large-scale and long-term studies are needed to validate this method from a clinical point of view.

## Competing interests

The author(s) declare that they have no competing interests.

## Authors' contributions

LG conceived the study, participated in its design and drafted the manuscript. KB performed the analysis and interpretation of the data and reviewed the manuscript. BS and RV performed the analysis and interpretation of the data, reviewed the manuscript and participated in the design of the study. GC and PA conceived the study and reviewed the manuscript. All authors read and approved the final manuscript.
